# Role and research progress of hip arthroscopy in the treatment of pre-collapse osteonecrosis of the femoral head

**DOI:** 10.3389/fsurg.2025.1603229

**Published:** 2025-12-05

**Authors:** Guangnian Liu, Xing Chen, Zijing Weng, Huazhang Xiong, Liming Dong

**Affiliations:** Department of Joint Surgery, Affiliated Hospital of Zunyi Medical University, Zunyi, China

**Keywords:** arthroscopy, distraction, treatment, femoral head, osteonecrosis

## Abstract

**Background:**

This study is to provide a detailed overview of the application value of hip arthroscopy in the treatment of pre-collapse ischemic necrosis of the femoral head (ONFH) and summarizes the latest research progress.

**Methods:**

Using the search string [(“osteonecrosis of the femoral head” OR “avascular necrosis of the femoral head” OR ONFH) AND (hip arthroscopy OR arthroscopic)] in PubMed, we identified 69 records. After screening and exclusions, 54 studies were included in a comprehensive narrative review of ONFH and hip arthroscopy. The review focused on the application of hip arthroscopy in core decompression and head–neck fenestration grafting for ONFH, and summarized the management of concomitant intra-articular lesions and arthroscopy-related complications.

**Results:**

As a novel technique applied in the early stages of ONFH, hip arthroscopy remains in an exploratory phase. Current literature affirms its clinical effectiveness in treating early ONFH, highlighting advantages such as highly precise positioning, shorter surgical duration, rapid postoperative recovery, and minimal surgical trauma compared to traditional approaches. However, challenges arise due to the unclear surface landmarks for arthroscopic entry, leading to a prolonged learning curve and demanding a high level of technical expertise from surgeons. Simultaneously, satisfactory outcomes have been achieved in addressing intra-articular pathologies accompanying the hip joint, such as femoroacetabular impingement, synovitis, and joint effusion.

**Conclusions:**

This review provides a systematic description of the role of hip arthroscopy in ONFH and summarizes the latest clinical applications of hip arthroscopy and its shortcomings.

## 1 Background

Osteonecrosis of the femoral head (ONFH) remains a challenging issue in young patients (1). Despite extensive scholarly research, the pathophysiological mechanisms of this condition remain unclear, and its underlying etiology is not fully elucidated. Previous studies have identified several risk factors, including steroid use, alcohol consumption, trauma, as well as a history of hypercoagulability and metabolic disorders (2, 3). ONFH is typically categorized as pre-collapse and post-collapse stages and treatment option selection depends on the disease stage (4). The management of pre-collapse ONFH is still controversial, given the high activity demands in young patients with early femoral head necrosis. Various joint-preserving surgical strategies have been proposed, including core decompression, percutaneous drilling, vascularized and non-vascularized bone grafting, and bone resection, among others (5). In recent years, the implementation of arthroscopic surgery has become widespread and evolved into an effective approach for preserving the hip, especially in the early stages of ONFH in young patients. The application of arthroscopy expands the field of view in acetabular labrum, femoral head, acetabular cartilage surfaces, central pit, ligaments, synovium, and the periarticular space around the greater trochanter in surgreries, it forms the basis for the diagnosis and minimally invasive treatment of hip joint diseases (6) and can simultaneously address accompanying pathologies within the hip joint caused by early femoral head necrosis (7). With the increasing incidence of ONFH in the younger population and the demand for improved quality of life, there is a growing need for early joint-preserving treatments. The application of hip arthroscopy in ONFH patients enables physicians to make more accurate diagnoses and choose more beneficial treatment methods. Therefore, the purpose of this article is to review the application of hip arthroscopy in ONFH and evaluate its effectiveness as a complementary technique to traditional surgical approaches in specific situations.

## 2 Positioning and establishment of hip arthroscopy portals

The establishment of the entry approach is crucial for arthroscopic treatment of the hip joint. The hip joint, a large ball-and-socket joint in the human body, is surrounded by abundant neurovascular structures. The surrounding muscular tissue is thick and structurally complex, making it challenging to find the correct path for needle penetration into the joint space (8). Traditional core decompression procedures impose high technical demands on the operator, requiring repeated intraoperative usage of fluoroscopy with a C-arm to accurately place the puncture needle (9). Repeated fluoroscopy procedures not only increase the risk of radiation exposure and surgical site infections but also elevate the risk of nerve traction injuries due to prolonged traction (10).

### 2.1 Anterolateral portal (ALP)

The ALP is positioned 1–2 cm proximal to the apex of the anterior edge of the greater trochanter, projecting on the skin at the junction between the middle and lateral third of the line connecting the anterior superior iliac spine (ASIS) and the greater trochanter. During portal creation, the hip is maintained in 15° abduction and 15° internal rotation. Under C-arm fluoroscopic guidance, the instrument trajectory is confirmed to avoid acetabular penetration or sciatic nerve injury (11). As the initial portal, the ALP provides a comprehensive view of the anterior acetabular wall, the weight-bearing zone of the femoral head, and the capsule (12), and is particularly useful for early diagnosis of synovial proliferation and cartilage delamination (13). Advantages include broad visualization and a low operating risk, making it suitable for most ONFH cases (11). However, exposure of the posterior acetabular wall and posterior femoral head is limited, often necessitating adjunct portals; excessive traction may increase the risk of injury to the lateral femoral cutaneous nerve (14).

### 2.2 Proximal anterolateral portal (PALP)

The PALP is established 2–3 cm distal to the ASIS along the anterior border of the iliotibial band (15). Branches of the lateral femoral cutaneous nerve should be avoided; an outside-in technique is used with the working depth confined to the superficial layer of the capsule (16). For anterosuperior necrotic zones of the femoral head (JIC types A/B), the PALP offers a vertical viewing angle that facilitates precise debridement of necrotic bone (17). In combination with the ALP, it broadens exposure of the anterior capsule and labrum; reports in ONFH with concomitant labral injury describe repair success rates up to 89% (18). Benefits include reduced instrument conflict and an optimized working corridor for anterosuperior lesions; however, because the portal lies near the femoral nerve, improper handling may cause sensory disturbances, and efficiency is lower for posterolateral disease (15).

### 2.3 Distal anterolateral portal (DALP)

The DALP is located 2–3 cm distal to the greater trochanter along the femoral axis. Under C-arm guidance, a 3.0 mm Kirschner wire is used to create a bone tunnel directed toward the necrotic region (19). This portal is commonly used for core decompression via multiple low-speed drillings to reduce intraosseous pressure; a 5-year survival rate of 84.78% has been reported (20). Compared with other portals, it is less invasive (entry diameter <3 mm) with minimal disruption of bony architecture and allows simultaneous arthroscopic management of intra-articular pathology (21). However, because optimal drilling angles must be controlled within approximately 30°–45°, the learning curve is steep and imaging navigation is often required; outcomes are limited in extensive necrosis (Kerboul angle >240°) (22).

### 2.4 Anterior portal (AP)

The AP is created at the midpoint of the line between the ASIS and the pubic symphysis, adjacent to the lateral border of the sartorius. Hip flexion of about 30° is used to relax the capsule and avoid the femoral artery and nerve (23). Capsulotomy through this portal can lower intra-articular pressure and improve femoral-head perfusion; in combination with the ALP, it is effective for addressing labral tears, with reports of postoperative Harris Hip Score improvement to 85.1 ± 7.7 (24). This portal allows direct management of capsular hypertension and labral pathology and may shorten rehabilitation time; nonetheless, the working corridor is narrow with higher risk of instrument collision, and excessive capsulotomy may increase the risk of instability (25).

## 3 Application of hip arthroscopy in core decompression of the proximal lateral femoral head in ONFH

Current studies suggest that symptoms of pre-collapse ONFH arise from three factors: (1) elevated intraosseous pressure in the femoral head, which impairs blood circulation and causes ischemia–hypoxia of bone tissue, leading to pain; (2) synovial hyperplasia that induces synovitis, resulting in joint edema/effusion and increased capsular pressure; and (3) mechanical symptoms—such as snapping hip, stiffness, or instability—caused by acetabular labral tears and cartilage flap delamination (26). According to the staging system of the International Research Circulation Osseous (ARCO), patients in ARCO stage I, corresponding to early collapse, are typically recommended for core decompression to preserve the hip (27). Traditional core decompression procedures typically using C-arm fluoroscopy for guidance to reach the necrotic area for decompression which heavily rely on the experience and technique of the operator (8). This method, however, has drawbacks, including unstable surgical times, increased blood loss, frequent fluoroscopy, and significant tissue damage. Moreover, the risk of puncturing the cartilage during the procedure poses a higher risk of damaging nerves, vessels, and muscles (28). In recent years, hip arthroscopy-assisted core decompression has gained widespread use in the treatment of ONFH due to its advantages of high precision in positioning, shorter surgical times, and reduced surgical risks (7).

### 3.1 Arthroscopic localization

In contrast to traditional core decompression, which often relies solely on C-arm fluoroscopy to guide the needle to the necrotic area, arthroscopy provides direct visualization. This allows for precise observation of the distinct boundary between the necrotic bone in the femoral head and the normal bone tissue, facilitating accurate placement of the guiding needle within the necrotic area (7, 29). Through medium to long-term follow-up observations of patients treated with hip arthroscopy, satisfactory clinical outcomes and favorable prognoses have been consistently achieved (30). This success can be attributed to the precise localization guided by hip arthroscopy, which reduces the risks associated with repeated puncture attempts and positioning.

### 3.2 Arthroscopic monitoring of tunnel wall lesions

In the early stages of ONFH, establishing a bone tunnel through core decompression is an effective method for reducing intraosseous pressure in the femoral head (31). Traditional core decompression, after creating the bone tunnel, relies solely on the operator's tactile mechanical sense to use curettes in various directions to remove necrotic bone. However, this mechanical approach often results in the inadvertent removal of normal bone trabeculae and may even penetrate through the subchondral bone, causing damage to the articular surface (32). With the assistance of arthroscopy, the direct observation of lesion areas is available in the established bone tunnel. This enables the surgeon to visually assess whether the bone tunnel traverses the necrotic area. The distinct boundary between white sclerotic bone and brownish necrotic bone against normal bone provides a clear border for determining the direction and location of debridement (33). Compared to the traditional methods which rely on the operator's experience and tactile sense, arthroscopy offers a more intuitive approach, enhancing surgical efficiency and reducing the risk of blind operations leading to subchondral bone damage (31).

## 4 Application of hip arthroscopy in ONFH with head-neck fenestration grafting (light bulb technique)

The light bulb technique, introduced by Rosenwasser et al. (34), involves creating a bulb-shaped cortical window at the junction of the femoral head and neck, followed by scraping and removing necrotic bone. The technique is named for the bulb-shaped cavity that resembles a light bulb after clearing the necrotic bone. Previous literature reports a 68%-86% success rate in preserving the hip joint 2–10 years after the light bulb technique (35). Clinical outcomes vary mainly due to differences in surgeon experience, the extent of surgical trauma, and the thoroughness of necrotic bone removal. The key to success lies in effectively and precisely removing necrotic bone, restoring blood flow, and avoiding excessive removal of normal bone tissue, preventing insufficient support leading to femoral head collapse. Previous studies focused on modifying the surgical approach to adequately expose the femoral head-neck junction, such as the Watson-Jones approach (36), Direct Anterior Approach (DAA) (37), and SuperCapsular Hip Arthroscopy-Directed (SHD) light bulb technique (38). However, these approaches cannot completely avoid the trauma caused by surgery and its impact on femoral head blood supply. Additionally, they lack direct observation of the light bulb cavity, hindering precise necrotic bone removal. Guadilla et al. (39) reported three cases of early-stage ONFH treated with arthroscopic light bulb technique, achieving good clinical results at three months postoperatively. Similar results were validated by Hua-zhang Xiong et al. (40), showing continuous improvement in range of motion (ROM), Harris Hip Score (HSS), Hip Outcome Score-Activities of Daily Living (HOS-ADL), and iHOT-12 after arthroscopic light bulb technique. Two years postoperatively, imaging studies (MRI, CT, and x-rays) demonstrated excellent graft healing. In summary, hip arthroscopy combined with the light bulb technique provides a minimally invasive approach for removing necrotic bone and restoring blood flow in pre-collapse ONFH patients, avoiding excessive removal of normal bone tissue to provide effective support. However, there is currently limited clinical reporting on the hip arthroscopy combined with the light bulb technique, with a lack of comparison with traditional procedures and long-term follow-up and evaluation.

## 5 Managing concomitant lesions in the hip joint

### 5.1 Treatment of femoroacetabular impingement syndrome (FAI)

Femoroacetabular impingement syndrome (FAI) results from congenital developmental abnormalities of the hip joint, causing abnormal morphology of the acetabulum and proximal femur. This leads to impingement during hip flexion, causing labral tears and damage to the articular cartilage (41). Traditional open surgical procedures, such as open osteotomy, can effectively remove bone spurs resulting from repeated impingements between the acetabulum and femoral head (42). However, these procedures are associated with drawbacks such as significant trauma, prolonged recovery time, and suboptimal incision healing (6). In recent years, with the widespread adoption and advancement of hip arthroscopy, it has become a more visually efficient approach to address intra-articular pathologies of the hip joint compared to traditional open surgery (43). Wang Jiangtao et al. (44) conducted a retrospective analysis of 24 cases treated with arthroscopic acetabuloplasty for isolated pincer-type FAI. The results indicated that hip arthroscopy effectively alleviated pain of hip joint caused by pincer-type FAI, with improvements in hip joint Visual Analog Scale (VAS) scores and modified Harris Hip Score (mHHS) in the short term. Zhang Jiaguo et al. (45) retrospectively analyzed clinical data from 86 FAI patients who underwent arthroscopic labral repair, femoral cam reshaping, and capsular plication using the shoelace technique. The average follow-up was 50.7 ± 15.8 months. Compared to preoperative assessments, patients showed improvements in mHHS scores, UCLA scores, VAS scores, and satisfaction scores. The results of these studies suggest that hip arthroscopy for treating FAI can significantly alleviate pain and symptoms of restricted movement of hip joint. Both short-term and long-term outcomes demonstrate satisfactory clinical results in terms of pain relief and functional improvement.

### 5.2 Managing synovitis and joint effusion

Research by Gao (46) and Bashaireh (47) suggests that the prolonged postoperative pain and slow recovery in patients with osteonecrosis of the femoral head (ONFH) may be related to hip synovitis and the resulting joint effusion. Arthroscopic clearance of synovitis can reduce intra-articular pressure, alleviate joint swelling, thereby relieve pain, and shorten the time for hip joint function recovery (6). Huazhang Xiong et al. (48) found that arthroscopic irrigation and debridement of synovitis effectively reduce inflammation and fluid accumulation within the joint capsule, decreasing the elevated pressure caused by effusion. This improvement enhances blood supply to the femoral head, thereby reducing postoperative pain and limitations in hip joint mobility.

However, current reported methods for synovial clearance mainly involve the excision of pathological synovial tissue, with various techniques lacking a standardized procedure to ensure complete removal of the affected synovium. The thoroughness of synovial clearance and its relationship with the prognosis of ONFH patients are rarely reported. Hao-Qiang Song et al. (26) introduced the arthroscopic total synovial peel (ATSP) procedure, which involves the complete detachment of the synovial layer, sub-synovial layer, and fat layer in the gap between the synovial layer and muscle layer. This technique provides a detailed anatomical approach for the complete removal of pathological synovial tissue. While the application of this method in clearing synovitis in the hip joint has not been reported, it holds potential benefits for the prognosis of ONFH patients.

## 6 Complications associated with hip arthroscopy

### 6.1 Complications arising from traction during hip arthroscopy

During hip arthroscopy, traction is utilized to separate the femoral head from the acetabulum, creating space for the introduction of arthroscopic instruments. However, this can lead to soft tissue injuries related to traction or injuries caused by continuous compression in the perineal area (49). Xue Jing et al. (50) conducted a retrospective analysis of complications in 365 patients undergoing hip arthroscopy. They found that among the 365 patients undergoing their first hip arthroscopy, no major complications occurred. The incidence of minor complications was 19.18%, with perineal, lower limb, and dorsum pedis numbness accounting for 5.75%, cartilage injury for 4.93%, labral penetration for 2.19%, and foreign body-related complications for 2.74%. As traction affects the entire leg during surgery, it may also lead to complications in the knee and ankle joints, but the statistical results are limited by the definitions of major and minor complications as defined by Kowalczuk (51) and Harris (52). Lone Frandsen et al. (53) conducted a prospective study on 100 patients treated with hip arthroscopy and found that 74% of patients reported traction-related symptoms postoperatively. Approximately 32% of patients experienced symptoms in the groin area, 49% reported symptoms in the knee joint, and 37% reported symptoms at the ankle joint with traction boot. This study identified a higher proportion of complications in the knee and ankle joints which has not been reported previously, but these complications are usually temporary and often disappear within 2–4 weeks as part of the postoperative recovery.

### 6.2 The impact of hip arthroscopy on total hip arthroplasty in late-stage femoral head osteonecrosis

Femoral head osteonecrosis is a progressive condition, and some patients who have undergone hip arthroscopy may still require total hip arthroplasty as the disease progresses. Therefore, understanding the impact of prior hip arthroscopy on subsequent total hip arthroplasty on the same side is crucial for patient decision-making, prognosis, and perioperative management. Nicholas J. Lemme et al. (54) analyzed 1940 patients without a history of hip arthroscopy and 1940 patients with a history of hip arthroscopy on the same side who underwent total hip arthroplasty through propensity-matched, their results exhibited that the average time from hip arthroscopy to total hip arthroplasty was 1,127 days. Compared to patients without prior hip arthroscopy, those with a history of hip arthroscopy on the same side had an increased risk of hip dislocation, prosthesis loosening, and revision within 1 year postoperative. Additionally, the prosthetic survival rate was reduced at 4 years postoperatively for patients with a history of hip arthroscopy.

## 7 Conclusions

In conclusion, as a novel technique applied in the early stages of ONFH, hip arthroscopy remains in an exploratory phase. Current literature affirms its clinical effectiveness in treating early ONFH, highlighting advantages such as highly precise positioning, shorter surgical duration, rapid postoperative recovery, and minimal surgical trauma compared to traditional approaches. However, challenges arise due to the unclear surface landmarks for arthroscopic entry, leading to a prolonged learning curve and demanding a high level of technical expertise from surgeons. Simultaneously, satisfactory outcomes have been achieved in addressing intra-articular pathologies accompanying the hip joint, such as femoroacetabular impingement, synovitis, and joint effusion. Nonetheless, attention is warranted for complications arising from traction. Recent literature reports cases of traction-free hip arthroscopy achieving satisfactory results. However, these reports are constrained by small sample sizes, a lack of high-level evidence from evidence-based medicine, and limited long-term follow-up data. Despite these challenges, investigating methods to establish arthroscopic entry rapidly and accurately, along with precise surface landmarking, remains a potential research direction to enhance the accuracy and safety of entry establishment. Furthermore, discussions on strategies to mitigate complications arising from traction necessitate further exploration.
